# Prostatic Metastasis of Bilateral Metachronous Testicular Cancer: A Case Report and Review of the Literature

**DOI:** 10.7759/cureus.91998

**Published:** 2025-09-10

**Authors:** Christos Diamantopoulos, Sampson Panagiotidis, Evangelos Varelas, Dimitrios Thanatopoulos, Ananstasios Fentas

**Affiliations:** 1 Department of Urology, General Hospital of Katerini, Katerini, GRC; 2 Department of Urology, General Hospital of Veroia, Veroia, GRC; 3 Department of Urology, General Hospital of Edessa, Edessa, GRC; 4 2nd Urologic Department of Aristotle University of Thessaloniki, Papageorgiou General Hospital, Thessaloniki, GRC

**Keywords:** bilateral testicular cancer, germ cell tumor, pelvic exenteration, prostate metastasis, triple chemotherapy regimen

## Abstract

Testicular cancer is an uncommon malignancy that primarily affects young men. Most cases are germ cell tumors (GCTs), which may present as seminomatous or non-seminomatous types. Bilateral occurrence is unusual, often developing at different times. Typical metastatic sites include retroperitoneal lymph nodes, lungs, and mediastinum, whereas prostate involvement is exceptionally uncommon.

We report a 39-year-old man with a history of bilateral, metachronous testicular cancer treated initially with orchiectomy and chemotherapy for a right-sided mixed GCT, followed by orchiectomy for a left-sided non-seminomatous germ cell tumor (NSGCT). He presented seven years later with urinary symptoms, elevated tumor markers, and imaging that revealed a large pelvic mass infiltrating the prostate, seminal vesicles, and left ureter. Biopsy confirmed metastatic NSGCT with elements of embryonal carcinoma, teratoma, and yolk sac tumor. Classified as poor prognosis by the International Germ Cell Cancer Collaborative Group (IGCCCG), he underwent salvage chemotherapy with ifosfamide, paclitaxel, and cisplatin (TIP), followed by radical cystoprostatectomy with pelvic exenteration. At six months post treatment, he remained disease-free with normalized tumor markers.

Prostate metastasis from testicular GCTs is an extremely rare event, especially following bilateral, metachronous primaries. This case is unique in demonstrating recurrence with histological elements from both tumors. Aggressive multimodal treatment with TIP, followed by pelvic exenteration, achieved radiological and biochemical response.

## Introduction

Testicular cancer occurs in less than 1% of men. Despite this, it is the most common cancer among younger males [1]. Testicular cancer is divided into two major categories based on histological characteristics: germ cell tumors (GCTs), which account for 95% of cases, and non-germ cell tumors, comprising 5% [1]. The first category can be subdivided into carcinoma in situ (GCNIS), seminomatous germ cell tumors (SGCT), non-seminomatous germ cell tumors (NSGCT), and mixed tumors. Non-seminomatous tumors include teratoma (postpubertal type), embryonal carcinoma, choriocarcinoma, and yolk sac tumor (postpubertal type) [2]. Among patients with GCTs, bilateral disease is rare, with a prevalence of 3-5%. When bilateral disease occurs, metachronous presentation is more common (65%) compared to synchronous presentation. In metachronous disease, the average time to the appearance of the second tumor is eight years, often exhibiting a different histology than the first tumor [3].

Based on tumor marker values, such as alpha-fetoprotein (AFP), beta-human chorionic gonadotropin (β-hCG), and lactate dehydrogenase (LDH), and the tumor-node-metastasis (TNM) classification system, the Union for International Cancer Control (UICC) proposed three clinical prognostic stages of disease (clinical stages I, II, and III) [4]. Regarding metastatic disease prognosis, the International Germ Cell Cancer Collaborative Group (IGCCCG) categorizes patients into three prognostic groups: good, intermediate, and poor [5].

GCTs most commonly metastasize via the lymphatic system to retroperitoneal lymph nodes and less commonly to the mediastinum, lungs, and pelvic lymph nodes [6]. Metastasis to the prostate, either de novo or post-orchiectomy, is very rare, with only 12 cases described in the literature to date [7-18]. The objective of this report is to present a rare case of prostate metastasis relapsing after bilateral, metachronous testicular GCT, highlighting its successful management through a multimodal treatment approach. A comparison of medical approaches used in similar cases documented in the literature is also attempted.

## Case presentation

A 39-year-old male presented to the urology outpatient clinic with complaints of dysuria and urinary frequency lasting six months. The patient was not married and had no children. His medical history included a right radical orchiectomy in 2014 at age 29, with histology revealing a mixed tumor of SGCT and NSGCT with embryonal carcinoma components (pT1N0M0). The disease was staged as clinical stage I; preoperative tumor markers were unavailable (SX). As treatment, he received two cycles of bleomycin, etoposide, and cisplatin (BEP). He was advised of sperm cryopreservation before treatment, but he was not interested. Two years later, in 2016, he underwent left orchiectomy. Histological examination showed NSGCT with teratoma and carcinoma in situ (pT1N0M0). Preoperative markers were β-hCG at 112 mIU/mL, AFP at 238 ng/mL, and LDH at 458 U/L, with clinical stage I. He was followed by oncology until 2018.

Additional history included cannabis use during adolescence, current smoking, subclinical hepatitis C, and testosterone therapy for hypogonadism after the last orchiectomy. He had no history of cryptorchidia. Physical examination revealed a painless abdomen without peritonitis signs. The scrotum showed no recurrence, but digital rectal examination revealed a large, hard prostate involving both lobes. No lymphadenopathy was noted.

**Figure 1:**
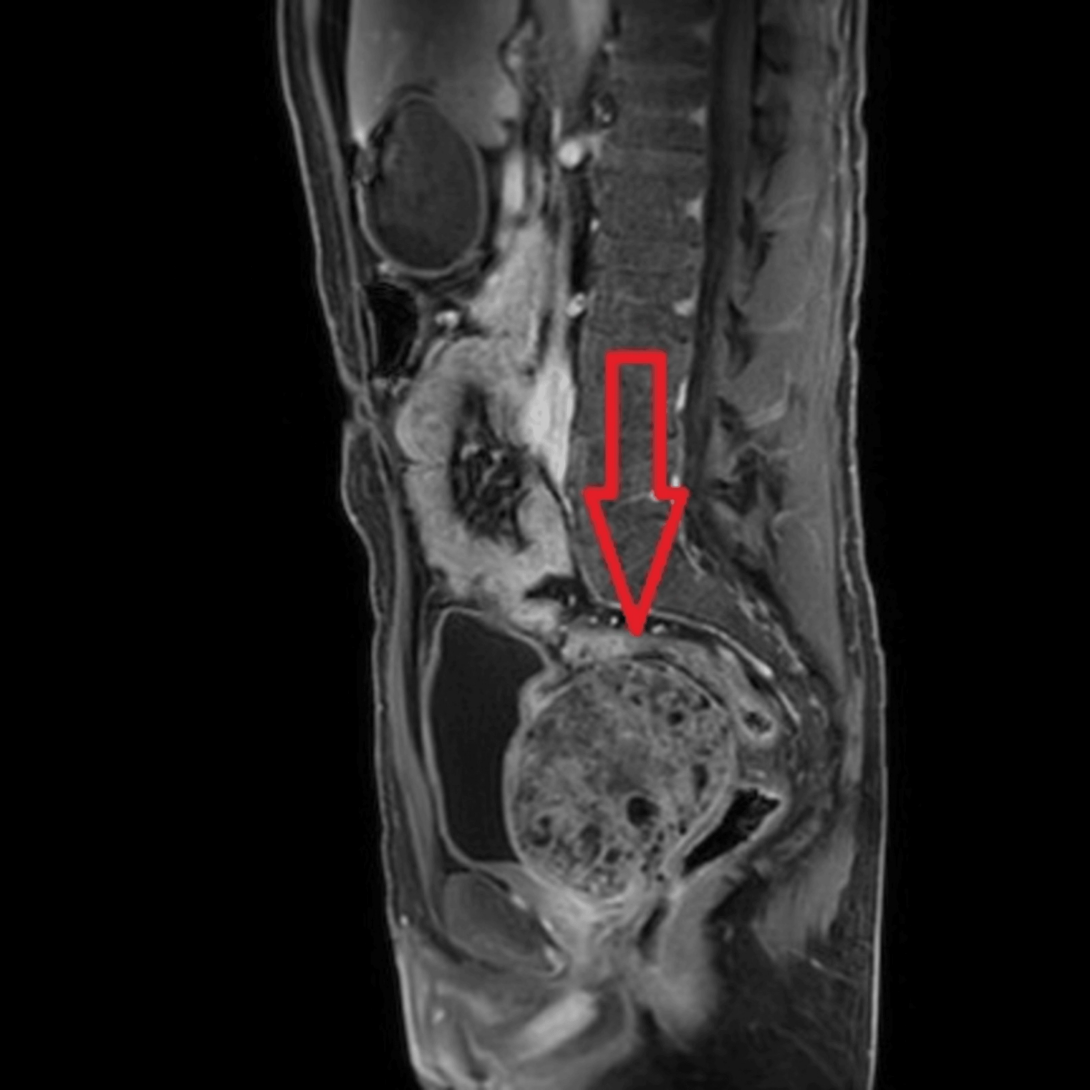
Abdominal MRI scan using the mDIXON method in sagittal plane showing sizable pelvic mass (arrow).

**Figure 2:**
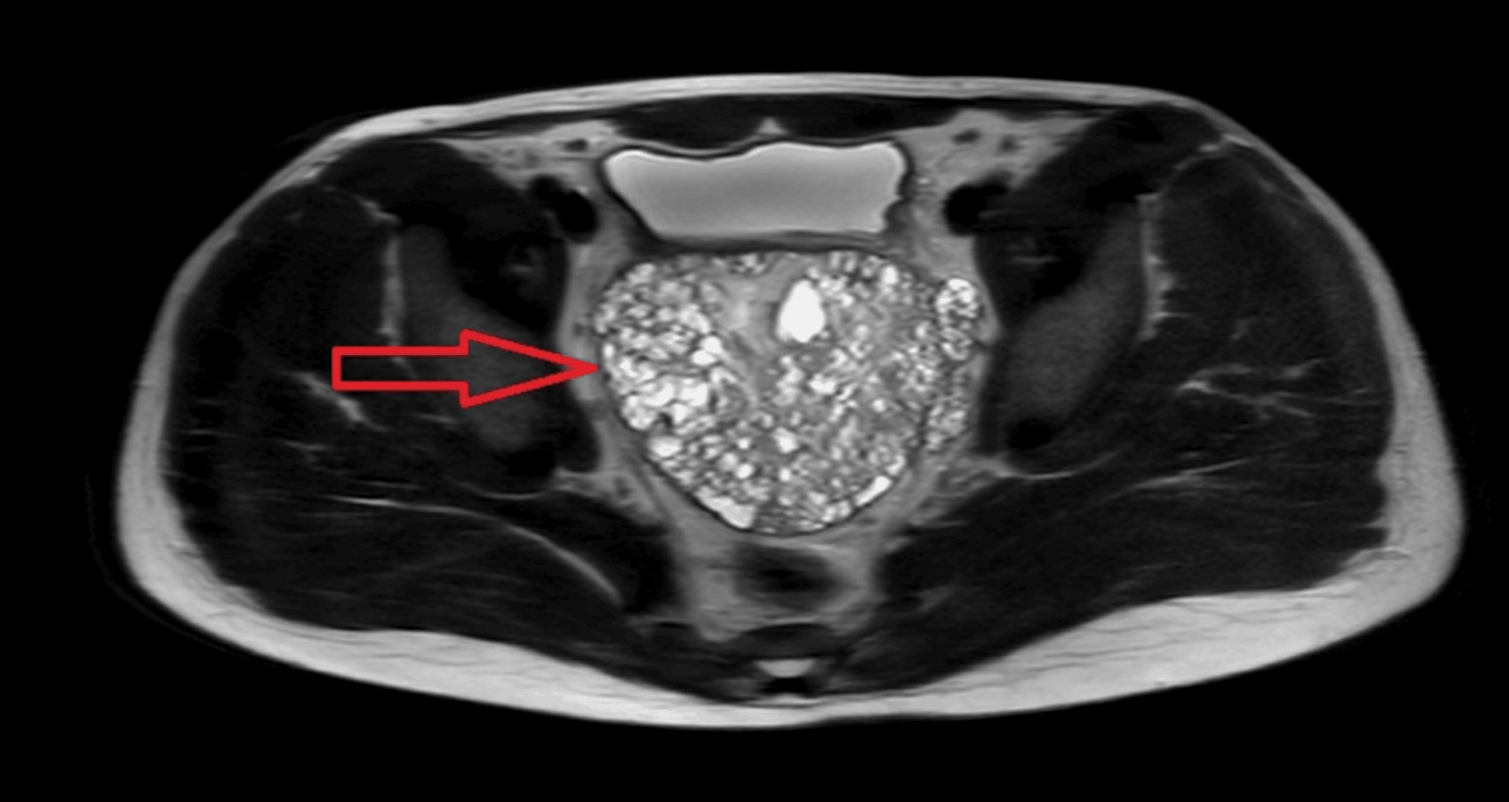
Abdominal MRI scan using T2-weighted turbo spin echo in the transverse plane showing a large, multicompartment, microcystic pelvic mass (101 x 75 x 86 mm) (arrow).

**Figure 3:**
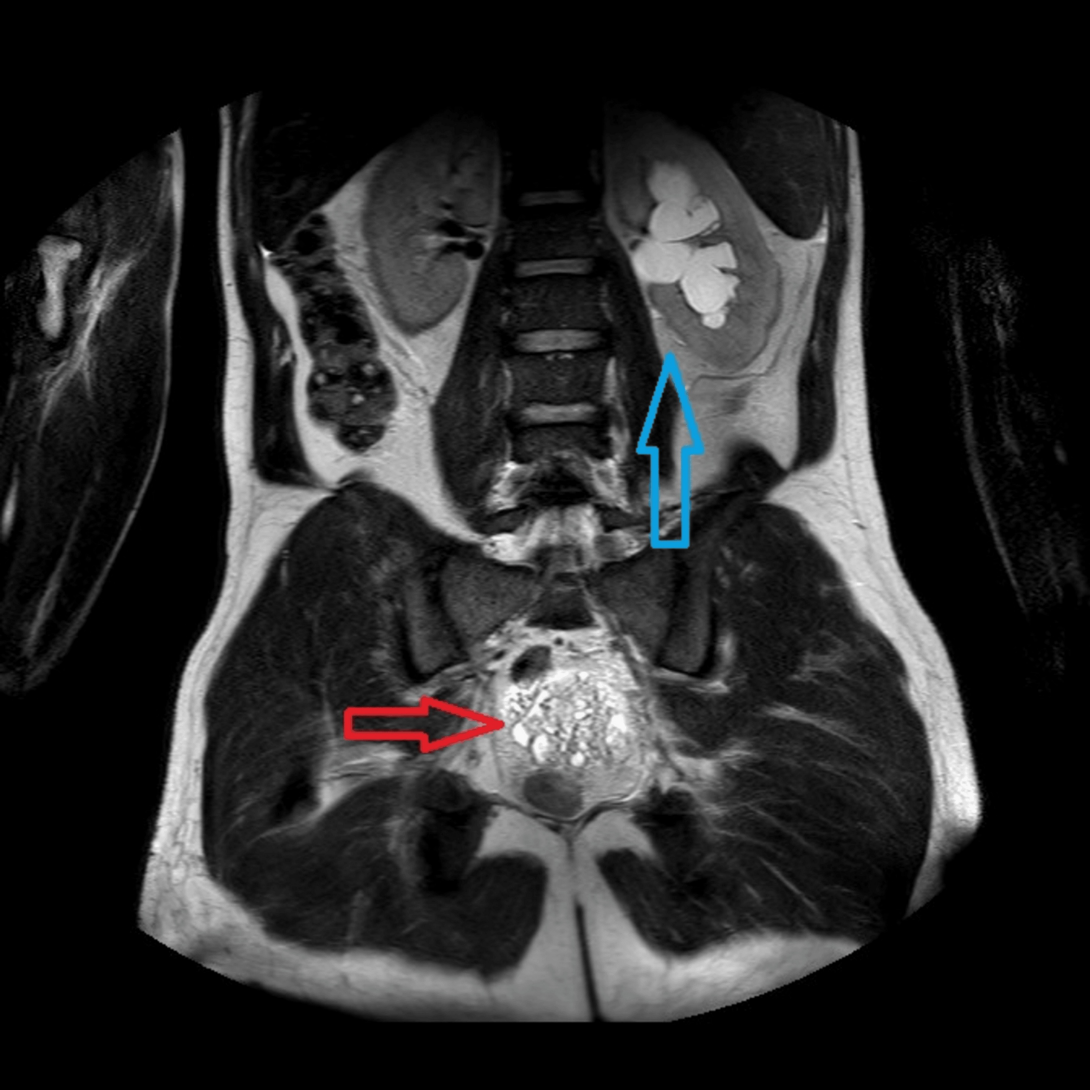
Abdominal MRI scan using T2-weighted turbo spin echo in the coronal plane showing neoplasm infiltrating the prostate (red arrow) and hydronephrosis of the left kidney (blue arrow).

Laboratory tests, including serum biochemistry and renal function, were normal. Tumor markers were as follows: β-hCG = 751.48 mIU/mL, AFP = 112.93 ng/mL, LDH = 160 U/L, and prostate-specific antigen = 0.61 ng/mL. A kidney-ureter-bladder ultrasound showed left hydronephrosis and a pelvic mass likely originating from the prostate. CT scan of chest and abdomen and MRI of the lower abdomen revealed a multicompartment, microcystic pelvic mass measuring 101 × 75 × 86 mm, infiltrating the prostate, seminal vesicles, and lower left ureter, causing hydronephrosis (Figures 1-3). The lesion displaced the bladder and rectum with signs of early infiltration (Figure 1). No visceral or lymphatic metastases were found.

Cystoscopy confirmed both ureteric orifices with no visible exophytic bladder lesions. Transrectal prostate biopsy (13 cores) revealed mixed NSGCT with embryonal carcinoma (corresponding to the first primary tumor), teratoma (corresponding to the second metachronous primary tumor), and yolk sac tumor components. Given the presence of visceral non-pulmonary metastasis, the patient was classified as poor prognosis by the IGCCCG criteria.

The oncology board decided on immediate chemotherapy with a triplet regimen: ifosfamide 1500 mg/m², paclitaxel 175 mg/m², and cisplatin 25 mg/m². Three cycles were administered every three weeks over 50 days, with mild side effects, such as weakness and fatigue. The patient subsequently underwent radical cystoprostatectomy with ileal conduit formation and low anterior resection with colostomy, performed jointly with general surgery.

**Figure 4:**
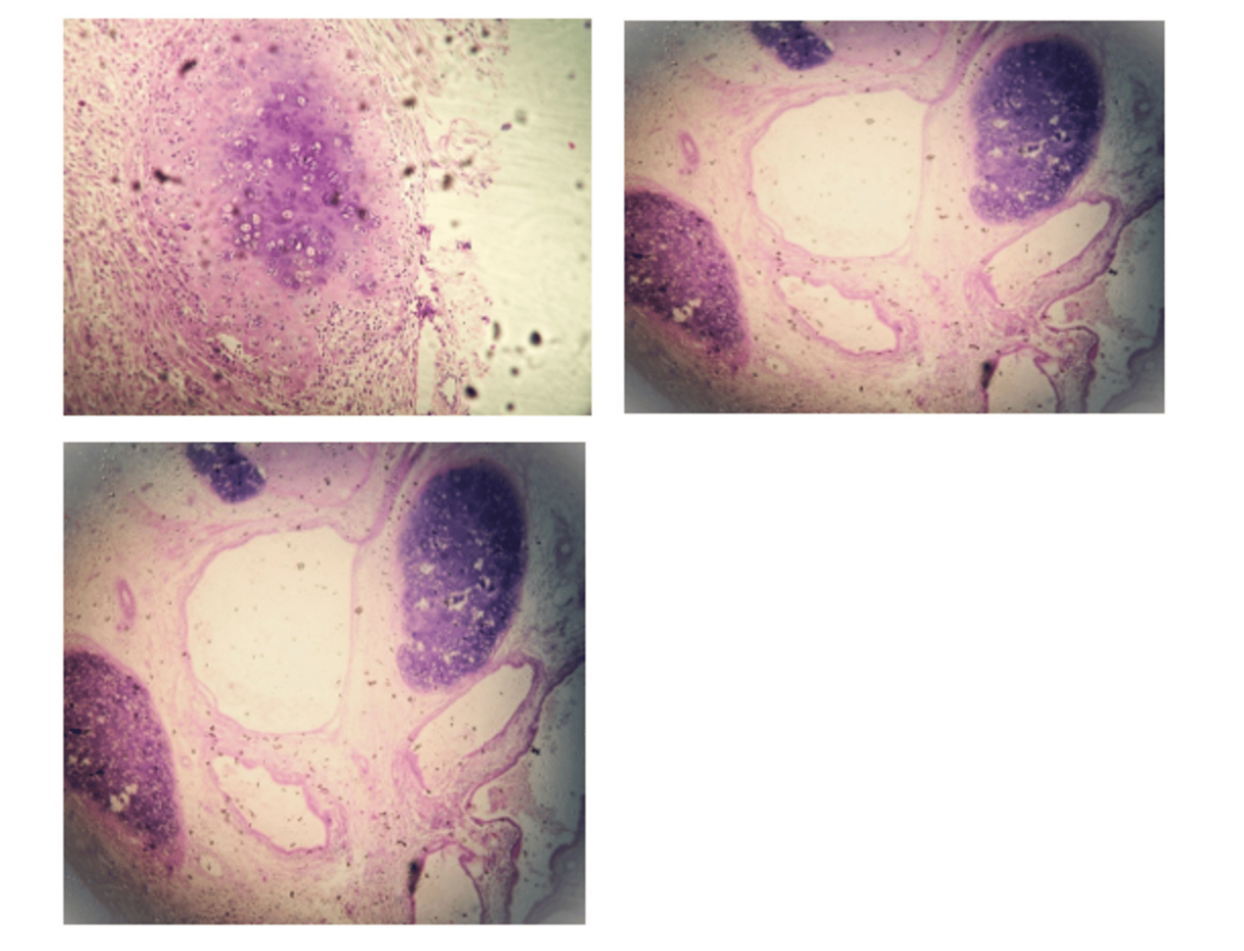
Pathological report. We observe infiltration of the prostate from parts of immature and mature cartilage tissue, as well as cystic spaces composed of mucinous or glandular-type epithelium.

Postoperatively, the patient developed a paralytic ileus managed conservatively. The hospital stay lasted 20 days. Final pathology showed NSGCT with teratoma features (Figure 4).

The follow-up of the patient is ongoing. The patient’s general condition is good, with one episode of upper urinary tract infection reported. Two months post surgery, tumor markers showed biochemical response: β-hCG = 2.8 mIU/mL, AFP = 2.39 ng/mL, and LDH = 158 U/L. At six months, CT of the thorax and abdomen showed no recurrence, and tumor markers were β-hCG at 2.4 mIU/mL, AFP at 1.9 ng/mL, and LDH at 173 U/L.

**Figure 5:**
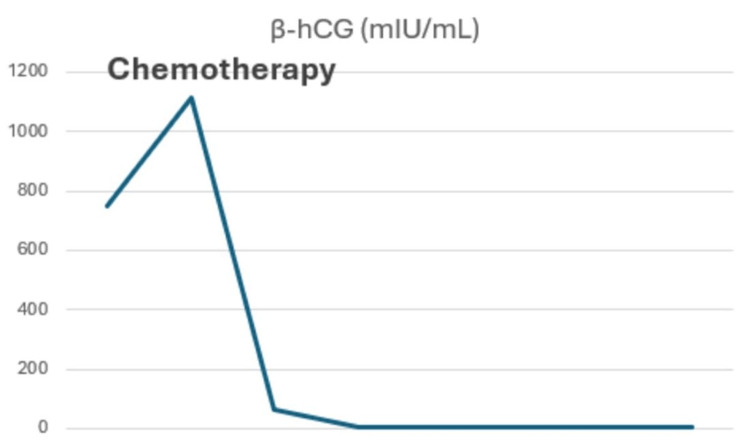
Process of beta-human chorionic gonadotropin (β-hCG) from the diagnosis of recurrence until now.

**Figure 6:**
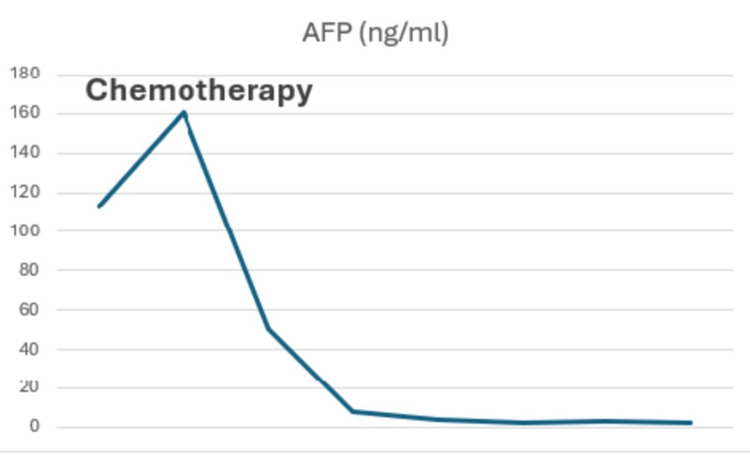
Process of alpha-fetoprotein (AFP) from the diagnosis of recurrence until now.

**Figure 7:**
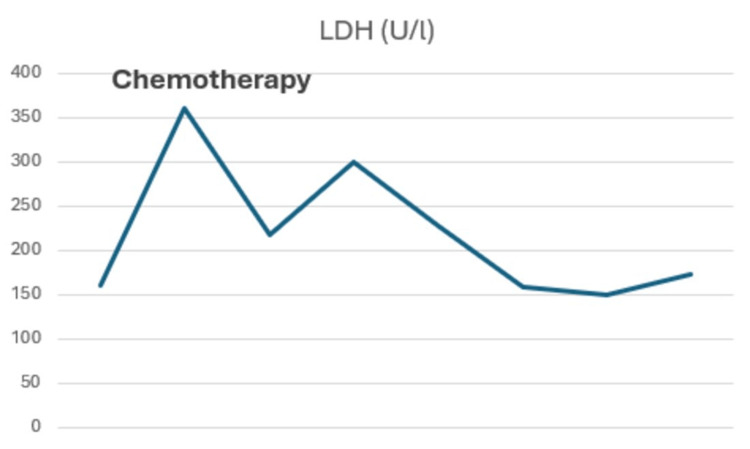
Process of lactate dehydrogenase (LDH) from the diagnosis of recurrence until now.

Table 1 and Figures 5-7 summarize tumor marker trends from diagnosis of recurrence to present.

**Table 1:** Changes in tumor marker levels from the moment of diagnosis of prostate metastasis until now. At week four, the patient started the triple chemotherapy regimen. At week 10, the patient underwent surgery. β-hCG: beta-human chorionic gonadotropin; AFP: alpha-fetoprotein; LDH: lactate dehydrogenase.

	Week 2	Week 4	Week 6	Week 8	Week 10	Week 12	Week 18	Week 24
β-hCG (<5 mIU/mL)	751.48	1113.1	64.7	3.1	2	2.8	2.4	2.4
AFP (<10 ng/mL)	112.93	160.68	50.28	7.65	4.18	2.39	3	1.9
LDH (140-180 U/l)	160	361	218	299	228	158	150	173

## Discussion

The patient was initially diagnosed with cancer of the right testis and underwent orchiectomy and chemotherapy (two cycles of BEP), based on the clinical stage and histological type. Histopathology revealed a mixed germ cell tumor with in situ carcinoma and elements of SGCT. Two years later, recurrence occurred in the contralateral testis.

The necessity of performing a contralateral testicular biopsy at initial diagnosis remains controversial. Data indicate the presence of germ cell neoplasia in situ (GCNIS) in up to 8.7% of contralateral testes [19], with malignant transformation occurring in approximately 50% of cases within five years [20]. Based on this, a contralateral biopsy was not performed at the time of the initial diagnosis.

Upon recurrence in the left testis, the patient underwent radical orchiectomy. Histopathology revealed a different subtype, and the disease was staged as clinical stage I NSGCT. Given the histological difference and disease-free interval, it was classified as a metachronous primary tumor. Consequently, the patient was managed with active surveillance without adjuvant chemotherapy. Literature reports relapse rates in clinical stage I NSGCT at approximately 17% within five years [21,22]. Most relapses occur within two years; only 4% relapse beyond that, which is considered a late relapse [21].

Seven years after treatment of the second tumor, the patient presented with a prostatic metastasis. Preoperative pathology suggested a mixed tumor with both SGCT and NSGCT elements. This raised the hypothesis of recurrence from both primary tumors, a rare phenomenon not widely described in the literature. However, the final pathology report post chemotherapy confirmed only NSGCT elements. In the literature, similar cases include nine with SGCT and four with NSGCT metastases to the prostate [7-18]. Recurrence rates differ by histology: 4-20% for SGCT and approximately 19-30% for NSGCT [21-24]. Visceral metastases are also more common in NSGCT (>15%) compared to SGCT (>5%) [25].

Among reported cases, four presented with prostate metastasis at initial diagnosis, while others developed it at relapse [7,8,14,15]. Four patients had isolated prostate recurrence, while others exhibited concurrent retroperitoneal or inguinal lymph node involvement [10,12,15,17]. Only one case had pulmonary metastasis [11]. These findings support the hypothesis of isolated tissue recurrence in the prostate, although further research is needed to substantiate this mechanism.

In terms of treatment, Baunacke et al. performed radical cystoprostatectomy in a patient previously treated with radiotherapy [12]. Farnham et al. managed a stage III tumor with BEP and orchiectomy, followed by radical cystoprostatectomy and etoposide, ifosfamide, and cisplatin (VIP) chemotherapy for recurrence; notably, prostate involvement existed at initial diagnosis [15]. Lesko et al. reported active surveillance after orchiectomy, with prostate recurrence managed with VIP combination [17]. Alsharif et al. described locally advanced prostate metastasis, although surgical details were lacking [10]. Our case is unique in that pelvic exenteration was performed for prostatic metastasis. Only two other published cases describe exenteration, both involving de novo prostate metastasis [26,27]. The main statements of this aggressive treatment are based on the young age of the patient, the oncologic burden, and the presence of signs and symptoms such as dysuria and hydronephrosis.

Regarding chemotherapy, our patient had a poor prognosis according to the IGCCCG criteria, with a five-year progression-free survival rate of 54% and overall survival of 67% for NSGCT [5]. While Durer et al. and Plummer et al. administered BEP in the presence of nodal disease [16,18] and Sagalowsky et al. used vinblastine, cisplatin, and bleomycin with pelvic radiation for retroperitoneal involvement [9], Lesko et al. treated recurrence with VIP [17]. Our patient received a unique regimen of ifosfamide, paclitaxel, and cisplatin (TIP). This combination has shown promising response rates, approximately 50%, in patients with poor prognosis and relapsed GCT following BEP treatment [28].

## Conclusions

This case highlights a rare presentation of testicular GCT with metachronous bilateral involvement and late recurrence manifesting as isolated prostate metastasis, an extremely uncommon event with only a few cases reported in the literature. Notably, our patient exhibited histological elements from both the initial and second primary tumors within the metastatic lesion, suggesting a unique pattern of disease progression. The initial biopsy reported the unique possibility of a metastasis containing elements from both primary tumors; however, the final pathology confirmed only elements of the second NSGCT. This may indicate a differential response to chemotherapy or sampling error in the initial biopsy, but the initial presentation was highly suggestive of this rare phenomenon. The use of salvage chemotherapy, followed by extensive surgical management, including pelvic exenteration, led to favorable short-term outcomes with no evidence of disease recurrence after six months. This case underscores the importance of long-term surveillance in patients with testicular cancer, especially those with bilateral or metachronous disease, and demonstrates that aggressive, multimodal therapy can be effective even in poor-prognosis scenarios. Further studies are warranted to better understand the mechanisms and optimal management strategies for such rare metastatic patterns.
